# Predicting accidental drug overdose as the cause of fatality in near real-time using the Suspected Potential Overdose Tracker (SPOT): public health implications

**DOI:** 10.1186/s12889-022-13700-0

**Published:** 2022-07-08

**Authors:** Karli R. Hochstatter, Sonal Rastogi, Kathryn Klein, Cameron Tait-Ozer, Nabila El-Bassel, Jason Graham

**Affiliations:** 1 Columbia University School of Social Work, New York, NY USA; 2 Friends Research Institute, Inc, Baltimore, MD USA; 3 New York/New Jersey High Intensity Drug Trafficking Area, New York, NY USA; 4 New York City Office of Chief Medical Examiner, New York, NY USA; 5 New York University Grossman School of Medicine, New York, NY USA

**Keywords:** Overdose death, Overdose surveillance, Overdose prediction tool, Opioids

## Abstract

**Background:**

Effective responses to the worsening drug overdose epidemic require accurate and timely drug overdose surveillance data. The objectives of this paper are to describe the development, functionality, and accuracy of the Suspected Potential Overdose Tracker (SPOT) for predicting accidental drug overdose as the cause and manner of death in near real-time, and public health implications of adopting the tool.

**Methods:**

SPOT was developed to rapidly identify overdose deaths through a simple and duplicable process using data collected by death investigators. The tool assigns each death a ranking of 1 through 3 based on the likelihood of it being an unintentional drug overdose, with 1 representing the highest likelihood that the death will be confirmed as an unintentional drug overdose and 3 representing the lowest. We measured the accuracy of the tool for predicting overdose deaths by comparing potential overdose deaths in New York City from 2018–2020 that were identified using SPOT to finalized death certificates. We also calculated the proportion of death certificate-confirmed overdoses that were missed by the SPOT tool and the proportion of type 1 errors.

**Results:**

SPOT captured up to 77% of unintentional drug overdose deaths using data collected within 72 h of fatality. The tool predicted unintentional drug overdose from 2018 to 2020 with 93–97% accuracy for cases assigned a ranking of 1, 87–91% accuracy for cases assigned a ranking of 2, and 62–73% accuracy for cases assigned a ranking of 3. Among all unintentional overdose deaths in 2018, 2019, and 2020, 21%, 28%, and 33% were missed by the SPOT tool, respectively. During this timeframe, the proportion of type 1 errors ranged from 15%-23%.

**Conclusions:**

SPOT may be used by health departments, epidemiologists, public health programs, and others to monitor overdose fatalities before death certificate data becomes available. Improved monitoring of overdose fatalities allows for rapid data-driven decision making, identification of gaps in public health and public safety overdose response, and evaluation and response to overdose prevention interventions, programs, and policies.

**Supplementary Information:**

The online version contains supplementary material available at 10.1186/s12889-022-13700-0.

## Introduction

The Centers for Disease Control and Prevention has estimated that more than 100,000 people in the U.S. died from drug overdose during the 12-month period ending in April 2021, a record high and nearly 30% increase from the previous year [1]. Over the past decade, the U.S. has experienced significant increases in drug overdose deaths, particularly involving synthetic opioids such as illicitly manufactured fentanyl [2]. Existing public health tools and funding for public health interventions have not kept pace with the urgency of this crisis.

Effective responses to the drug overdose epidemic require accurate and timely drug overdose surveillance data [3]. Death certificates are the foundation of overdose mortality surveillance [4]. However, long turnaround times for issuing finalized death certificates in suspected drug-related deaths hinders surveillance activities and prevents rapid responses by public health programs. For example, determining whether a death is due to drug overdose often requires a forensic autopsy and toxicology analysis, and may involve obtaining information from public officials, family and friends [5]. Once the cause of death has been determined, death certificates go through several steps prior to becoming available for analysis and statistical reporting [5]. For drug overdose deaths, on average 37.8% of death certificate records are available for analysis in the National Center for Health Statistics’ database by 13 weeks (1 quarter), 82.7% by 26 weeks (2 quarters), and 95.0% by 39 weeks (3 quarters) [5]. The length of lag time, from when the death occurs to when the jurisdiction completes the death certificate, is longer for overdose deaths than for all other injury-related deaths combined [5]. In addition to lengthy autopsy, toxicology, and other data collection techniques required for suspected drug-related deaths, timely and accurate reporting of overdose mortality data is complicated by regional variations in coding and the rapidly evolving nature of the opioid overdose crisis [5]. To identify geographic regions at risk of high overdose mortality and improve public health preparedness and prevention efforts, predictive modeling approaches have been developed [6–8]. However, the timeliness of data availability shapes the utility of these methods. Given the rapidly changing nature of the opioid overdose epidemic, [9] better and more timely data on overdose deaths are needed to enable rigorous analyses of the drug overdose crisis and provide greater predictive benefit. As adulteration of the drug supply continues to increase, [10] rapid data on overdose mortality would allow for the detection of localized “spikes” and quickly alert authorities to particularly dangerous changes in the drug supply (e.g., counterfeit pharmaceuticals; contamination of a stimulant supply with fentanyl; presence of a new high-potency synthetic opioid in the drug supply, etc.). Improved monitoring of overdose fatalities through a simple and duplicable process will allow for data-driven decision making and the identification of gaps in public health and public safety overdose response preparedness. Additionally, quick and accurate identification of accidental drug overdose deaths will offer opportunities to evaluate overdose prevention interventions, programs, and policies.

Medicolegal death investigations are initiated as required by state laws in cases of unexplained, sudden, and/or unnatural deaths, including suspected intentional or unintentional drug overdose deaths [4, 11]. Medical examiners and coroners, which vary across jurisdictions in their selection (appointed or elected) and professional credentialing, [12, 13] and other death investigators, play a critical role in generating data for the medical certification section of death certificates [11, 14]. While the National Association of Medical Examiners (NAME) provides evidence-based recommendations for investigating and certifying deaths associated with opioids and other drugs to improve death certificate data, [15] there are currently no nationally-accepted best practices, standards, or guidelines for using these improved data to monitor overdose fatalities before death certificate data becomes available. Death investigation data are routinely collected immediately after the overdose fatality occurs and provide an opportunity to rapidly examine factors and circumstances of overdose mortality and conduct time-sensitive activities.

The need for more timely and actionable information on overdose fatalities prompted the New York City (NYC) Office of Chief Medical Examiner (OCME) to develop a tool that uses data generated during death investigations to identify drug overdose deaths in near real-time. This tool, named the Suspected Potential Overdose Tracker (SPOT), uses a small number of variables that can be easily and rapidly collected, followed by a rigorous and reproducible algorithm to identify accidental drug overdose fatalities. The purpose of this paper is to describe: 1) the development and functionality of the SPOT tool, 2) the accuracy of the tool for predicting accidental drug overdose as the cause and manner of fatality, and 3) public health implications of the SPOT tool for reducing overdose deaths.

## Methods

### Setting

This paper examines unintentional overdose deaths in NYC. The NYC OCME is the largest medical examiner’s office in the country [16] From 2010 to 2020, the rate of overdose deaths in New York City (NYC) increased from 8.2 per 100,000 to 30.5 [17]. In 2020, opioids were involved in 85% of overdose deaths in NYC; fentanyl was present in 77% [17]. The OCME defines unintentional overdose deaths by an accidental manner of death and a cause of death ascribed to acute intoxication by substances that include but are not limited to prescription or illicit opioids, benzodiazepines, stimulants, and other central nervous system depressants. This definition of unintentional overdoses excludes poisonings where the manner of death is classified as intentional (suicide), undetermined, or homicide, and where the only substances listed were alcohol and/or marijuana.

### Data sources

To determine whether a case meets the criteria for a suspected unintentional drug overdose, the SPOT tool relies on data captured at the death scene along with any relevant case documents produced during the investigation. Data sources include, but are not limited to, the medicolegal investigation report, police reports, clinical information from hospitals, toxicology and autopsy reports, and narrative reports from families, friends, or other peers. Collectively, these data make up a case file for each individual. Case files are stored on a secured server at the OCME. Death certificates, which are typically the last report uploaded, are also included in each individual’s case file.

### Development of the SPOT Likelihood Scale

The SPOT tool was created using key variables identified through a series of comprehensive statistical analyses and team discussions. During development, the goal was to simplify, improve replicability, increase accuracy, and capture as many overdoses as possible. The tool was developed using the OCME case file data from 2018. The process began by using linear and logistic regression models to examine whether each variable captured by the OCME was associated with a death certificate being finalized that concluded the death was an unintentional drug overdose. Over 50 variables were examined (see Additional file 1 for a list of variables considered for inclusion in the SPOT tool). Variables that were most strongly associated with overdose death and available for the greatest number of cases were retained in the final model. Development of the SPOT tool was an iterative process of data-driven adjustments to refine and improve performance of the scale, resulting in a set of strict and well-defined criteria to inform the review and identification of each suspected drug overdose death. This paper describes and examines the final model that had the highest accuracy and captured the most unintentional overdose deaths. The final SPOT tool assigns each suspected overdose case to one of three categories, which vary in their probability that the death will be confirmed as an accidental drug overdose death. Having multiple categories provides an improved understanding of how likely it is that each case was an overdose death, allowing public health officials to use the information and intervene differently depending on this level of certainty. While four- and five-category models were tested during development, the three-category model better met the goals of the tool, as described above.

### Variables Included in the SPOT Tool

The SPOT tool uses the following key variables that are available for most suspected drug-related deaths: age; manner and cause of death (pending further study or already certified by day of review); case-specific details surrounding circumstances of the death; hospital toxicology testing (blood or urine) positive for presence of drugs prior to any hospital administered medications (e.g., benzodiazepines, opioids, cocaine), excluding alcohol and marijuana; any mention or knowledge of substance use history, specified or unspecified, excluding alcohol and marijuana; any mention of prior overdose; evidence of any paraphernalia associated with substance use found on scene at location of injury; and evidence of substance use prior to death that is often determined through visual assessment, whether through witness observation or clear evidence in immediate vicinity of the body. This information is extracted from each case file and entered into a SPOT investigation template (Additional file 2).

### The SPOT Likelihood Scale

Using all available investigative data captured in the case file, investigators apply specific criteria to each case and assign a ranking of 1 through 3, with 1 representing the highest likelihood that the death will be confirmed as an unintentional drug overdose and 3 representing the lowest. A small number of cases can be confirmed as an overdose due to availability of a finalized death certificate within 24–72 h of the death and are given the designation of ‘OD’, separate from the 1–3 rankings. Cases are assigned the confirmed OD designation if there is a finalized death certificate the day of review confirming that the cause of death is an acute intoxication due to substance(s), includes at least one substance of interest (opioid, stimulant, central nervous system depressant, phencyclidine, or ketamine), and the manner of death is accidental. Cases receive a likelihood ranking of 1 if a witness confirmed drug use or there was evidence in the immediate vicinity of the body suggesting drug use prior to death. A likelihood ranking of 2 is assigned if there was on scene evidence of any paraphernalia associated with substance use, but not suggestive of use immediately prior to death, or the decedent had a history of prior overdose. Lastly, cases receive a likelihood ranking of 3 if the decedent was ≤ 65 years of age and either had a prior history of substance use (excluding alcohol or marijuana) or a hospital toxicology test was positive for relevant drugs. Criteria for these rankings are further specified in Additional file 3, which provides the inclusion and exclusion criteria, definitions, and process flow. Rankings are not mutually exclusive. If a case meets the criteria for more than one ranking, it is assigned the ranking with the highest likelihood of being an unintentional drug overdose (i.e., lowest numerical value).

### Evaluation of the SPOT tool

All deaths in NYC from 2018 that fell under medical examiner jurisdiction and were taken to the OCME for postmortem examination were comprehensively reviewed by investigators. Cases that appeared unlikely to be caused by drug overdose (e.g., cases of apparent or determined suicide, homicide, trauma, infant death, etc.) based on an initial assessment of each case file were filtered out, and the SPOT tool criteria were applied to all remaining possible overdose cases. Each case received a ranking of OD, 1, 2, or, 3. For each ranking category, the proportion of cases confirmed to be unintentional overdose fatalities was calculated (i.e., the positive predictive value), as determined by finalized death certificates. Cases that met SPOT exclusionary criteria (see Additional file 3) or did not meet criteria for OD, 1, 2, or 3 were excluded. The proportion of overdose deaths captured by the SPOT tool (i.e., the sensitivity), overall and for each ranking, was also calculated by dividing the number of deaths captured by the total number of death certificate-confirmed unintentional overdose deaths in NYC. To examine how the tool performed beyond 2018, we applied the SPOT criteria to all possible overdose deaths in 2019 and 2020 using the same processes. For each year and likelihood ranking, we also calculated the proportion of death certificate-confirmed overdoses that were missed by the SPOT tool (i.e., the type 2 error rate) and the proportion of cases identified by the SPOT tool as suspected drug overdoses that later had finalized death certificates confirming the cause of death to be undetermined or a cause other than accidental drug overdose (i.e., the proportion of false-positives or type 1 errors). Analyses were conducted using Stata/SE 16.1 (College Station, TX).

## Results

### Sociodemographic characteristics

Table 1 presents demographic and drug use characteristics cases identified as possible drug overdose deaths when applying the SPOT algorithm to 2018 death data (the population used to develop the SPOT tool). Among these 1125 confirmed drug overdose cases, the average age was 46 years, 862 (77%) were male, 414 (37%) were White, and 278 (25%) were Black or African American. The most common drugs decedents had a history of using included heroin and cocaine.

**Table 1:** Demographic and Drug Use Characteristics Among Overdose Decedents Identified using SPOT: New York City, 2018

Characteristic	Confirmed OD (*n* = 29) n (%)	Ranking 1 (*n* = 200) n (%)	Ranking 2 (*n* = 556) n (%)	Ranking 3 (*n* = 627) n (%)
Age				
mean, st. dev	51, 10.4	42, 13.8	46, 13.3	49, 11.1
Gender				
Male	18 (62)	139 (70)	455 (82)	476 (76)
Female	11 (38)	61 (31)	101 (18)	151 (24)
Race/Ethnicity (n, %)				
White	9 (31)	70 (36)	253 (46)	165 (27)
Black/African American	14 (48)	45 (23)	116 (21)	231 (37)
Hispanic/Latino	6 (21)	70 (36)	161 (29)	207 (33)
Asian/Pacific Islander	-	8 (4)	12 (2)	12 (2)
Other/Unknown	-	-	-	7 (1)
Drug Use History^a^				
Any	27 (93)	168 (84)	434 (78)	595 (95)
Alcohol	9 (31)	33 (17)	125 (22)	209 (33)
Marijuana	-	12 (6)	53 (10)	63 (10)
Phencyclidine (PCP)	-	-	-	7 (1)
Heroin	11 (38)	54 (27)	146 (26)	123 (20)
Cocaine	14 (48)	41 (21)	72 (13)	135 (22)
Crack	-	16 (8)	61 (11)	72 (11)
Oxycodone	-	-	24 (4)	15 (2)
Hydrocodone	-	-	-	-
Alprazolam	-	-	19 (3)	11 (2)
Benzodiazepine or Zolpidem	-	-	15 (3)	14 (2)
Methamphetamine	-	12 (6)	8 (1)	11 (2)
Methadone	-	-	-	-
Opiate NOS	-	12 (6)	46 (8)	60 (10)
Prescription Opioid	-	8 (4)	49 (9)	47 (8)
History of Intravenous Drug Use^a^				
Yes	-	38 (19)	62 (11)	66 (11)

*OD*   Overdose, *NOS*  Not otherwise specified

Values of n ≤ 5 were suppressed for confidentiality

^a^Drug use history is likely significantly under reported because use of substances not mentioned in the case file are unknown

### Overdose cases captured using the SPOT tool

Among all deaths in NYC falling under medical examiner jurisdiction and brought to the OCME for postmortem examination, 2733 were considered possible overdose cases and underwent evaluation using the SPOT tool in 2018, compared to 2929 in 2019 and 2479 in 2020. The reduced number of cases in 2020 was likely a result of the change in criteria for which decedents were brought to the OCME for postmortem examination during the COVID-19 pandemic, which reduced the number of cases transported to the OCME. Among these, 1321, 1547, and 734 cases were excluded in 2018, 2019, and 2020, respectively, because they met SPOT exclusionary criteria or did not meet criteria for likelihood rankings 1, 2, or 3 and were therefore the least likely to be unintentional overdose fatalities. Table 2 presents the number of cases that met criteria for confirmed OD and likelihood rankings 1, 2, and 3. In 2018, 2019, and 2020: 96%, 93%, and 97% were confirmed as unintentional overdose deaths among cases with a ranking of 1; 90%, 87%, and 91% were confirmed among cases with a ranking of 2; and 65%, 62%, and 73% were confirmed among cases with a ranking of 3, respectively. Overall, the 3-category SPOT tool was 79%, 77%, and 85% accurate (i.e., positive predictive value) at determining whether a death was an unintentional drug overdose in 2018, 2019, and 2020, respectively.

**Table 2:** Number of suspected accidental overdose cases identified and the accuracy of SPOT, New York City 2018–2020

	n	A. Number of suspected overdose cases identified by SPOT	B. Number of suspected overdose cases identified by SPOT that were confirmed by death certificate	C. Positive Predictive Value (B/A)	D. Number of suspected overdose cases identified by SPOT that were either confirmed to not be accidental overdoses or were undetermined (A-B)	E. Proportion of false positives (D/A)	F. Proportion of all overdoses (B or n /total N)
**2018**							
Overall^a^	-	1383	1096	79%	287	21%	77%
Ranking 1	-	200	192	96%	8	4%	14%
Ranking 2	-	556	498	90%	58	10%	35%
Ranking 3	-	627	406	65%	221	35%	29%
Confirmed OD^b^	29	-	-	-	-	-	2%
Missed by SPOT	292	-	-	-	-	-	21%
**Total N**^**c**^	**1417**	-	-	-	-	-	**100%**
**2019**							
Overall^a^	-	1369	1060	77%	309	23%	71%
Ranking 1	-	217	201	93%	16	7%	13%
Ranking 2	-	584	506	87%	78	13%	34%
Ranking 3	-	568	353	62%	215	38%	24%
Confirmed OD^b^	13	-	-	-	-	-	1%
Missed by SPOT	418	-	-	-	-	-	28%
**Total N**^**c**^	**1491**	-	-	-	-	-	**100%**
**2020**							
Overall^a^	-	1724	1464	85%	260	15%	66%
Ranking 1	-	287	279	97%	8	3%	13%
Ranking 2	-	770	697	91%	73	9%	31%
Ranking 3	-	667	488	73%	179	27%	22%
Confirmed OD^b^	21	-	-	-	-	-	1%
Missed by SPOT	733	-	-	-	-	-	33%
**Total N**^**c**^	**2218**	**-**	**-**	**-**	**-**	**-**	**100%**

*OD* Overdose, *SPOT* Suspected Potential Overdose Tracker

^a^Excludes confirmed OD cases

^b^Number with a finalized death certificate at the time of implementing the SPOT tool (within 72 h of the death)

^c^Total N is the number of death certificates that were issued in NYC finalizing the manner and cause of death as unintentional drug overdose

A total of 1417, 1491, and 2218 death certificates were issued in NYC certifying the cause and manner of death as unintentional drug overdose in 2018, 2019, and 2020, respectively. Of these, 77%, 71%, and 66% were captured each year using the SPOT tool (i.e., sensitivity). Again, a lower proportion of deaths were likely captured in 2020 due to the change in criteria for which cases were brought to the OCME for postmortem examination during the COVID-19 pandemic. The proportions captured among each likelihood ranking are presented in Table 2.

### Confirmed overdose cases missed by the SPOT tool

Table 2 presents how many finalized overdose deaths were missed by SPOT (i.e., false negatives or type 2 errors). Among all confirmed unintentional overdose deaths in 2018, 2019, and 2020, 21%, 28%, and 33% were missed by the SPOT tool, respectively.

### Cases identified by the SPOT tool that were not confirmed overdose cases

Among all cases captured by the SPOT tool and assigned a likelihood ranking in 2018, 2019, and 2020, 21%, 23%, and 15%, respectively, had finalized death certificates confirming the cause of death to be undetermined or a cause other than accidental drug overdose (i.e., false positives or type 1 errors). These false positives are likely overestimated because a portion of the “undetermined” deaths are likely to be accidental overdose deaths.

## Discussion

The SPOT tool allows for rapid prediction of unintentional overdose as the cause of death and achieves a benchmark of identifying up to 77% of total drug overdose fatalities in a major U.S. city. The strict and well-defined criteria predicted unintentional drug overdoses from 2018 to 2020 with 93–97% accuracy for cases assigned a ranking of 1, 87–91% accuracy for cases assigned a ranking of 2, and 62–73% accuracy for cases assigned a ranking of 3. The tool used data collected during death investigations within 72 h of the fatality.

State and county public health departments are increasingly implementing web-portals, such as opioid overdose surveillance dashboards in California, [18] Rhode Island, [19, 20] Michigan, [21] and New York state, [22] where preliminary data are made publicly available on a quarterly, biannual, or near-real time basis. While these dashboards have used data collected by medical examiners and medicolegal death investigators to determine suspected overdose deaths, [21, 23, 24] algorithms for determining suspected overdose cases remain inconsistent and unknown, and many systems continue to rely on delayed death certificate data. This is the first paper to examine and describe a replicable tool for determining accidental drug overdose as the cause of death. Key strengths of the SPOT tool include the replicability, ability to make timely predictions, algorithm simplicity that public health workers and other professionals can be quickly trained on, and high accuracy. The tool also uses data elements that several state and national professional associations recommend collecting during potential drug-related death investigations. Thus, these data are often recorded routinely by coroners and medical examiners. The universality of SPOT datapoints will ease implementation efforts in other jurisdictions and allow the tool to be continually evaluated. The high performance of the SPOT tool during 2020 also demonstrates its reliability under suboptimal circumstances, while medical examiner offices dealt with the overwhelming increase of deaths due to the COVID-19 pandemic. As funding for enhanced and timely surveillance of drug overdose increases, [25] states and counties may apply the SPOT algorithm to their enhanced surveillance data for improved overdose monitoring and rapid data dissemination and response in their specific locality.

### Limitations

Reporting on suspected overdose deaths determined using the SPOT tool is not 100% accurate. Depending on how the data are used, caution must be used to avoid any potential risks of false positives. Additionally, the overdose epidemic may continuously evolve as new risks or substances are introduced to the drug supply. Examples of temporal factors that may affect performance of the SPOT tool include the widespread adulteration of the illicit drug supply with fentanyl, the increasing prevalence of overdoses involving psychostimulants, a growing proportion of the population having experienced a prior overdose, and the expanding availability of harm reduction interventions such as naloxone distribution and safe injection facilities. Thus, it is important to routinely evaluate the predictive value of each criterion, reassess the accuracy of the tool, and make refinements if necessary. Additionally, there may be drug use subcultures and overdose characteristics (e.g., spaces of use, routes of administration, and socio-legal context of use) unique to other jurisdictions that are not present in NYC. Thus, it is important to assess the performance of the tool when deployed in different geographic regions of the country. Lastly, implementation of the SPOT tool requires significant investment by the agency that collects the data in its current form. Resource availability may vary greatly across jurisdictions and outside funding sources may be required for financial support. Advanced data extraction and reporting techniques that provide automation of SPOT would significantly improve the usability of SPOT, particularly in low-resourced coroner and medical examiner settings.

## Public health implications

The SPOT tool may be used by health departments, epidemiologists, public health programs, and others in a multitude of ways, such as reporting and data sharing. Community and health policy makers can use the data from SPOT to inform a rapid deployment of evidence-based interventions to reduce overdose deaths. The findings from the tool may also drive case-level investigations and inform the allocation of resources for jurisdictions that have limited death investigation resources.

### Reporting and data sharing

Epidemiologists or other public health personnel may analyze overdose cases identified through the SPOT tool and present key findings routinely (e.g., daily, weekly, etc.), informed by which information recipients deem most relevant and actionable. These reports may include analyses of suspected overdose fatalities for the preceding month stratified by likelihood ranking, decedents’ demographic characteristics, and general (unidentifiable) location of overdose. When available, information regarding decedents’ employment status, insurance status, prior substance use, participation in drug treatment, existence of co-occurring health conditions, and criminal justice involvement may also be presented. These reports may be tailored and distributed to representatives and leaders from various partner agencies, including public health and safety partners that are most likely to intersect with people at risk of overdose. The disclosure of any case-specific information should be limited to the minimum necessary to accomplish the intended purpose of the requesting agency. Partner agencies may use the data to better understand the incidence of drug overdose fatalities within the population they serve and evaluate their own prevention efforts. Waiting for completed death certificates has the potential to delay rapid responses to the growing fentanyl epidemic affecting many communities, [10] and particularly in communities of color [26]. These reports represent a novel model for sharing death investigation data with external agencies. All suspected drug overdose cases reported using the SPOT tool should be continually reviewed and updated to reflect final cause and manner of death information.

### Case-level investigations

The SPOT tool can be used to further investigate the circumstances and psychosocial factors contributing to individual suspected overdose fatalities. For example, social workers or case managers may identify and interview social network members of suspected overdose decedents ranked 1 by the SPOT tool, among possible others, and collect critical historical and contextual information that further informs partners’ efforts to reduce overdose fatalities. This would enable death investigation teams to provide pertinent information for jurisdiction-wide fatality review, connect grieving family members to grief and bereavement services, and refer decedents’ network members to addiction treatment and harm reduction services using peer navigation or other evidence-based intervention strategies.

### Resource allocation

NAME recommends a forensic autopsy examination whenever intoxication is suspected as a possible cause for death [15]. NAME and The American College of Medical Toxicology also recommend performing comprehensive toxicology testing for most suspected drug overdose cases [27]. The surge in overdose deaths has challenged already strained death investigation teams, who often face budget constraints and have limited capacity to perform autopsies and toxicology testing. For example, medicolegal death investigation offices require a pathologist for each additional 250 to 325 cases per year [28]. By identifying cases with a high likelihood of being confirmed unintentional overdose deaths, the SPOT tool may be used in low resource settings to reduce the autopsy and toxicology caseload and allocate resources to cases with a lower likelihood of being a confirmed overdose death; that is, to those cases in which there is less certainty regarding cause and manner of death for which autopsy and/or comprehensive toxicology testing may be more critically needed.

## Conclusions

The SPOT tool can predict accidental drug overdose with high accuracy, allowing for improved monitoring of overdose fatalities through a simple and duplicable process using data collected immediately or otherwise very early during death investigations. The public health implications of adopting this tool are of high impact: 1) it allows for near real-time monitoring of overdose fatality levels and detection of atypical patterns jurisdiction-wide, as well as within specific geographic areas and populations; 2) it provides a mechanism for expedited outreach to family and friends of overdose decedents who may be at high risk for overdose; 3) it allows for identification of gaps in overdose response preparedness and data-driven decision making of public health and public safety agencies; and 4) it affords opportunities for evaluation and consistent information exchange of overdose prevention interventions, programs and policies being implemented by participating public health and public safety agencies.

## Supplementary Information

**Additional file 1.** Variables considered during development for inclusion in the SPOT tool.

**Additional file 2.** SPOT data collection template.

**Additional file 3.** SPOT likelihood scale.

## Data Availability

The datasets generated and analyzed during the current study are not publicly available due to privacy and ethical concerns but are available from the corresponding author on reasonable request. Administrative permissions were not required to access the raw data. The raw data was not shared outside of the OCME.

## Abbreviations

SPOT: Suspected Potential Overdose Tracker
NYC: New York City
OCME: Office of Chief Medical Examiner
NAME: National Association of Medical Examiners
