# Supplementary material for: Predicting accidental drug overdose as the cause of fatality in near real-time using the Suspected Potential Overdose Tracker (SPOT): public health implications
Source: BMC Public Health. 2022 Jul 8;22:1311. doi: 10.1186/s12889-022-13700-0 (PMC9263436; doi:10.1186/s12889-022-13700-0)
Supplement: Supplementary file 1 — Additional file 1. Variables considered during development for inclusion in the SPOT tool. [file 12889_2022_13700_MOESM1_ESM.pdf]

Data Dictionary

**REPORT DATE**	This is the date on which OCME team is pulling case census of deaths from previous calendar day; Monday through Friday.
**RESEARCHERS**	This is the last name of the individual(s) who were working on the cases. The first name listed is the individual who typed the data in Excel. This field is helpful if there are questions on a case and there are variations in team schedules.
**LIKELIHOOD**	OD = This incident already had a finalized death certificate the day of review confirming that it is an unintentional acute drug intoxication (MOD: accidental, COD: "acute intoxication by substance(s)"<br>1 = Witness confirming drug use or evidence in immediate vicinity of body suggesting substance drug use prior to death.<br>2= Decedent found with drugs or paraphernalia in immediate vicinity or decedent had history of prior overdose<br>3 = Decedent has prior history of substance use; or, hospital toxicology was positive for relevant drugs; decedent must also be 65 years of age or under
**OCME NUMBER**	Unique OCME case number assigned to each decedent
**DOD**	Date of death
**HOSPITAL ADMIT DATE**	Most recent date on which decedent was admitted to hospital prior to death (if applicable and available).
**NYPD NOTIFICATION**	An accountability notification system specific to OCME that tracks whether NYPD was aware of a suspected overdose and present on scene of investigation, or was notified of suspected overdose by OCME personnel if not aware and absent from scene.
**FIRST NAME**	Decedent's first name and middle if provided
**LAST NAME**	Decedent's last name
**DOB**	Date of birth
**AGE**	Approximate age of decedent on date of death [auto-filled via the following Excel function: (Date of death-date of birth)/365.25]
**RACE**	Race as reported in NYC OCME's CMS.<br>W = Non-Hispanic white<br>B = Non-Hispanic black<br>H = Hispanic<br>A/P = Asian/Pacific<br>O = Other<br>U = Unknown<br>AN = American Native

**GENDER**	Gender as reported in NYC OCME's CMS.<br>M = Male<br>F = Female<br>TM = Transgender Male<br>TF = Transgender Female
**OCME NYSID**	If decedent has a New York State Identification Number (NYSID), then it is included here. This information is found in case notes.<br>NO NYSID = No fingerprint match<br>PRINT NOT RUN = Fingerprint not submitted for matching<br>UNKNOWN = No information
**NYPD NYSID**	Only New York State Identification Number (NYSIDs) for NYC residents are retained for this field
**JUSTICE SYSTEM INVOLVEMENT**	Justice system-related history included here in narrative form, if available.
**NOTES**	Any pertinent notes not captured in remainder of spreadsheet. Recorded in narrative form.
**PHYSICAL HEALTH**	Physical health conditions and history mentioned in MLI narrative report or case notes
**MENTAL HEALTH**	Name of psychiatric disorders mentioned, including suicide attempts and ideations. Include unspecified and undiagnosed as well.
**SUBSTANCE USE TX**	If MLI report has any mention of previous or current treatment history (include when if stated)
**PRIOR OD**	If decedent had a history of prior overdose, and number of prior overdoses, if mentioned
**PRESCRIPTIONS**	Relevant prescriptions that the decedent is believed to use, either through evidence of what is found on scene, or through determining recent prescription history based on available medical records.
**NALOXONE**	If naloxone was administered, by who, and how many doses<br>EMS/ED = Naloxone administered by medical professionals (EMS, including FDNY/ED)<br>FF = Naloxone administered by friends/family/someone who knew decedent<br>NYPD = Naloxone administered by NYPD<br>STAFF = If Naloxone administered by staff (transitional housing, treatment housing etc.)<br>UNKNOWN = Administrator not identified; however, noted in MLI report or police report that it was administered (Also use “Unknown” if potentially self-administered since it is difficult to positively ascertain that the decedent self-administered naloxone.)
**HX_UNSPECIFIED**	1 = Unspecified substance use history/polysubstance use history
**HX_IVDA**	1 = Any mention of intravenous drug use (IVDA) method of drug use in MLI report or case notes

**HX_ HEROIN**	1 = Any mention of heroin use in MLI report or case notes
**HX_COCAINE**	1 = Any mention of cocaine use in MLI report or case notes
**HX_CRACK**	1 = Any mention of crack cocaine use in MLI report or case notes
**HX_AMPHETAMINE**	1 = Any mention of Methamphetamine / Amphetamine (Adderall) / MDMA (Ecstasy) use in MLI report or case notes
**HX_KETAMINE**	1 = Any mention of ketamine use in MLI report or case notes
**HX_PCP**	1 = Any mention of PCP use in MLI report or case notes
**HX_OPIOID**	1 = Any mention of Opiate / Opioid use in MLI report or case notes
**HX_BENZO**	1 = Any mention of Benzodiazepine use in MLI report or case notes
**HX_OTHER_RX**	1 = Any mention of non-opioid, non-benzodiazepine or non-amphetamine prescription medication use other than as-prescribed in MLI report or case notes
**HOSPITAL TOXICOLOGY**	1 = Decedent tested positive for drugs during hospital toxicology (blood or urine sample) test as per MLI report or ED physician
**S_COCAINE**	1 = If hospital toxicology test was positive for cocaine as per MLI report or ED physician. *Note: tests do not distinguish between powder cocaine and crack cocaine*
**S_OPIOID**	1 = If hospital toxicology test was positive for opiate(s) / opioid(s) as per MLI report or ED physician
**S_BENZO**	1 = If hospital toxicology test was positive for benzodiazepine(s) as per MLI report or ED physician
**S_AMPHETAMINES**	1 = If hospital toxicology test was positive for amphetamines(s) / methamphetamine / MDMA as per MLI report or ED physician
**S_OTHER**	1 = If hospital toxicology test was positive for category of drug other than those specifically listed as per MLI report or ED physician
**GLASSINES**	1 = Glassines, plastic baggies, Ziploc bags, or wax paper envelopes that appear to be used for drugs found on scene as per MLI report or case notes
**GLASSINE DESCRIPTION**	Glassine color(s), stamp(s) or distinctive marking(s), etc.
**POWDER**	1 = Any powder found on scene as per MLI report or case notes
**STRAW**	1 = Straw used for insufflation found on scene as per MLI report or case notes
**SYRINGE**	1 = Syringe(s) found on scene as per MLI report or case notes
**GLASS PIPE**	1 = Glass pipe(s) found on scene as per MLI report or case notes (exclude if only marijuana-related)
**SPOON**	1 = Spoon with substance residue found on scene as per MLI report or case notes
**PILLS**	1 = Loose/unlabeled pills found on scene as per MLI report or case notes

**ADDL_PARA_DETAIL**	Description of any other drug-related paraphernalia not otherwise captured in paraphernalia categories on spreadsheet
**DEATH ADDRESS**	Street number and address of death location as reported in CMS, MLI report, or police report<br>UNKNOWN = If death location address is unknown
**DEATH APT**	Apartment number if reported in CMS, MLI report, or police report<br>UNKNOWN = If death location address is unknown
**DEATH CITY**	City of death as reported in CMS, MLI report, or police report<br>OUT OF CITY = Death location address is out of NYC<br>UNKNOWN = If death city is unknown
**DEATH STATE**	State of death as reported in CMS, MLI report, or police report<br>OUT OF STATE = Death location address is out of NYC<br>UNKNOWN = If death state is unknown
**DEATH ZIP**	Zip code of death as reported in MLI report or police report<br>UNKNOWN = If death zip code is unknown
**DEATH LOC TYPE**	Type of death location as reported in MLI report or police report. Ex. Decedent residence, other residence, medical facility, restaurant, subway, etc.
**DEATH PCT**	Death precinct as reported in MLI report, police report, or by searching through NYPD online precinct finder *(https://www1.nyc.gov/site/nypd/bureaus/patrol/find-your-precinct.page)*<br>OUT OF CITY = Death address is outside of NYC<br>OUT OF STATE = Death address is out of NYS<br>UNKNOWN = Death address is unknown. This could apply if the decedent is either unidentifiable and/or had no form of identification present.
**FORMULA (1=death address)**	1 = Decedent died at home
**HOME ADDRESS**	Street number and address of residence as reported in CMS, MLI report, police report, or case notes<br>UNKNOWN = Home address is unknown
**HOME APT**	Apartment number if reported in CMS, MLI report, or police report<br>UNKNOWN = If home address is unknown
**HOME CITY**	Home city as reported in CMS, MLI report, or police report<br>UNKNOWN = Home address is unknown
**HOME STATE**	Home state as reported in CMS, MLI report, or police report<br>UNKNOWN = Home address is unknown
**HOME ZIP**	Home zip code of death as reported in CMS, MLI report, or police report,<br>UNKNOWN = Home address is unknown
**HOME LOC TYPE**	Denote only for social services residences such as SRO/supportive housing/homeless shelter/NYCHA/assisted living, if applicable, as noted in MLI report

**HOME PCT**	Home precinct as reported in MLI report, police report, or by searching through NYPD online precinct finder<br>*(https://www1.nyc.gov/site/nypd/bureaus/patrol/find-your-precinct.page)*<br>OUT OF CITY = Home address is outside of NYC<br>OUT OF STATE = Home address is out of NYS<br>UNKNOWN = Home address is unknown. This could apply if the decedent is either unidentifiable and/or had no form of identification present.
**HOME PTRL BORO**	Home patrol borough as defined by home precinct (will autofill when HOME PCT column is filled)<br>OUT OF STATE = Home address is out of NYS<br>HOMELESS = Decedent has no known home address<br>UNKNOWN = Home address is unknown. This could apply if the decedent is either unidentifiable and/or had no form of identification present.
**FORMULA (1=death address, 2=home address)**	1=Decedent found at death location<br>2=Decedent found at home
**FOUND ADDRESS**	Street number and address of location where decedent was found as reported in MLI report or police report<br>UNKNOWN = No report of where decedent was found
**FOUND APT**	Apartment number if reported in MLI report or police report<br>UNKNOWN = If found location is unknown
**FOUND CITY**	City where found as reported in MLI report or police report<br>UNKNOWN = No report of where decedent was found
**FOUND STATE**	State where found as reported in MLI report or police report<br>UNKNOWN = No report of where decedent was found
**FOUND ZIP**	Zip code where found as reported in MLI report or police report<br>UNKNOWN = No report of where decedent was found
**FOUND LOC TYPE**	Location where found as reported in MLI report or police report<br>UNKNOWN = No report of where decedent was found
**FOUND PCT**	Precinct where found as reported in MLI report, police report, or by searching through NYPD online precinct finder<br>UNKNOWN = No report of where decedent was found
**FOUND PTRL BORO**	Patrol borough found as defined by precinct where decedent was found<br>UNKNOWN = No report of where decedent was found
**CMPLNT PCT**	Precinct of complaint number attached to case as per CMS (will autofill when FOUND PCT is filled)
**61**	NYPD complaint number attached to case as per CMS listed as 61 number
**CMPLNT**	Complete NYPD Complaint number (will autofill when 61 is filled)
**AIDED**	NYPD Aided Card identification number listed in CMS
**NYPD INVOICE**	Voucher numbers associated with different paraphernalia tagged on scene; later provided by NYPD
