# Supplementary material for: Predicting accidental drug overdose as the cause of fatality in near real-time using the Suspected Potential Overdose Tracker (SPOT): public health implications
Source: BMC Public Health. 2022 Jul 8;22:1311. doi: 10.1186/s12889-022-13700-0 (PMC9263436; doi:10.1186/s12889-022-13700-0)
Supplement: Supplementary file 2 — Additional file 2. SPOT data collection template. [file 12889_2022_13700_MOESM2_ESM.pdf]

NYC OCME Suspected Potential Overdose Tracker (SPOT) Investigation

**ID/Case #**	**Last Name**	**First Name**		
______	______	______		
**DOB**	**DOD**	**Age**	**Race**	**Gender**
______	______	______	______	______

Locations

Found Address ____________________ **Location Type** ______
Street address *City* *State* *Zip*

Death Address ____________________ **Location Type** ______
Street address *City* *State* *Zip*

Home Address ____________________ **Location Type** ______
Street address *City* *State* *Zip*

Investigation Questions

Is there evidence of substances or drug paraphernalia associated with substance use on scene? YES/NO

If yes, specify which and location

- ☐ POWDER ☐ GLASS PIPE ☐ GLASSINES
- ☐ STRAW ☐ SPOON *DESCRIPTION* ______
- ☐ SYRINGE ☐ PILLS ☐ OTHER ______

Can drug use <u>immediately</u> prior to death be determined through one of the following scenarios? YES/NO

A. Decedent was **seen or known** to use drugs according to friend, family, neighbor, etc.

B. Decedent **admitted** to using drugs prior to fatal event

C. Decedent was **seen** with clear evidence of recent drug use prior to fatal event. Examples include:

- *Belt/band/tourniquet tied around decedent's arm*
- *Syringe/needle in decedent's arm*
- *Powder on decedent's face, hands, air passages etc.*
- *Paraphernalia in decedent's hand*

Specify which scenario:

Does the decedent have a history of prior overdose? YES/NO

If yes, do you know number of times?

Does the decedent have a history of substance use (excluding alcohol and marijuana)? YES/NO

If yes, please list which substances:

- ☐ UNSPECIFIED ☐ COCAINE ☐ KETAMINE ☐ BENZODIAZEPINE
- ☐ IVDA ☐ CRACK ☐ PCP ☐ OTHER RX
- ☐ HEROIN ☐ AMPHETAMINE ☐ OPIOID

Any substance use treatment history? YES/NO

If yes, please list which treatment:

If death occurred at a hospital:

Did hospital toxicology tests report substances (excluding alcohol and marijuana)? YES/NO

If yes, please list which substances:

- ☐ COCAINE ☐ BENZODIAZEPINE ☐ OTHER
- ☐ OPIOID ☐ AMPHETAMINE

Additional information if applicable:

Was naloxone administered prior to death? YES/NO

If yes, by who and how many doses?

Relevant health history:

Relevant mental health history:

Relevant prescriptions:

Any other relevant case-specific circumstances surrounding fatal event:
