# Supplementary material for: Predicting accidental drug overdose as the cause of fatality in near real-time using the Suspected Potential Overdose Tracker (SPOT): public health implications
Source: BMC Public Health. 2022 Jul 8;22:1311. doi: 10.1186/s12889-022-13700-0 (PMC9263436; doi:10.1186/s12889-022-13700-0)
Supplement: Supplementary file 3 — Additional file 3. SPOT likelihood scale. [file 12889_2022_13700_MOESM3_ESM.pdf]

NYC OCME Suspected Potential Overdose Tracker (SPOT)

Likelihood Scale

Classifications are not mutually exclusive. A case may meet the criteria for more than one classification. However, always assign a classification value according to case criteria that meet the classification with the highest level of accuracy.

START — Identify each case in your jurisdiction in which a death investigation is required to adjudicate cause and manner of death

A small number of cases may be confirmed as an overdose with 24-72 hours of death

Higher suspected accuracy

OD *Confirmed*

If all of the following apply:
1. On finalized death certificate, cause of death includes "acute intoxication due to substance(s)[4]"
2. Includes at least one substance of interest[4]
3. Manner of death is Accidental

1 *Suspected*

If at least one of the following applies[5]:
1. Witness confirming substance use immediately prior to death
2. Clear evidence in immediate vicinity of body suggesting substance use prior to death

2 *Suspected*

If at least one of the following applies:
1. On scene evidence of any paraphernalia[6] associated with substance use
2. Any mention of prior overdose

3 *Suspected*

1. If age ≤ 65 years old AND at least one of the following applies:
    a. Any mention of substance use history[4] (excluding alcohol and marijuana)
    b. Hospital toxicology testing (blood or urine) positive[4] prior to any hospital-administered medications (excluding alcohol and marijuana)

Exclude if death certificate is finalized and does not meet criteria of overdose[2]

Exclude if case details indicate immediately exclusionary case circumstances or other MOD[3]

Exclude all cases with only alcohol- and marijuana-related paraphernalia found on scene

Exclude all other cases

Further information on the back.

[1] *Daily Case Census*

In NYC, a list of all fatalities that have been accepted by the OCME called the "Daily Case Census" is generated every 24 hours. This may vary or come from multiple sources in your jurisdiction.

[2] *Definition of overdose*

Manner of death:		Cause of death:
Accidental	**AND**	"Acute intoxication due to substance(s)"

[3] *For immediate exclusion*

- Manners of death (MOD)
    - Natural
    - Suicide
    - Homicide
    - Therapeutic complication
    - Undetermined
- Case circumstances
    - Obvious trauma (i.e. gunshot wound, stabbed, took a fall, motor vehicle accident, etc.)
    - Infants and toddlers (≤ 36 months)
    - Old age (≥ 75 years old) and/or extensive and severe medical history (HTN, COPD, CVA, HBP, MI, MVA, DM, CHF, cancer, etc.)
    - Found location is outside jurisdiction of interest

[4] *Categories/substances of interest (prescription or illicit)*

- Opioids
- Stimulants
    - Cocaine
    - Amphetamines/methamphetamines
- CNS Depressants
    - Benzodiazepines
    - Sleep drugs and tranquilizers
    - Muscle relaxants
    - Antipsychotics
- PCP
- Ketamine

[5] *Ascertaining substance use prior to fatal event*

- Drug use prior to death is often determined through visual assessment
    - Decedent was **seen** or **known** to use drugs according to friend/family/neighbor/medical professional etc.
    - Decedent **admitted** to using drugs prior to fatal event
    - Decedent was **seen** with clear evidence of recent drug use prior to fatal event. Examples include:
        - Syringe/needle in decedent's arm
        - Paraphernalia in decedent's hand
        - Belt/band/tourniquet tied around decedent's arm
        - Powder found decedent's face, hands, air passages etc.

Note: Paraphernalia found on decedent's person (ex. Paraphernalia in pockets) is **not** considered a category 1. This situation **does not** clearly indicate drug use immediately prior to death.

[6] *Paraphernalia associated with substance use*

- Syringe/hypodermic needle
- Syringe caps/hypodermic needle caps
- Baggies, glassines, zip lock bags, or wax envelopes
- White, brown, or black powder/substance
- Cotton
- Cords/belts/bands used to create tourniquets
- Spoon with residue
- Rolled bills, straws, or small tubes
- Tin/aluminum foil
- Glass pipes
- Loose, unlabeled controlled prescription drugs/pills
- Bottle caps

See the FDA Drug Safety Communications issued on 8-31-2016 and 9-20-2017 for additional information on opioids, benzodiazepines, and other central nervous depressants.
